# Inactivation of Lon protease reveals a link between mitochondrial unfolded protein stress and mitochondrial translation inhibition

**DOI:** 10.1038/s41419-018-1213-6

**Published:** 2018-12-05

**Authors:** Gautam Pareek, Leo J. Pallanck

**Affiliations:** Department of Genome Sciences, University of Washington, 3720 15th Avenue NE, Seattle, WA 98195 USA

## Abstract

The mitochondrial Unfolded Protein Response (UPR^mt^) pathway confers protection from misfolded and aggregated proteins by activating factors that promote protein folding and degradation. Our recent work on Lon protease, a member of the mitochondrial ATPase Associated with diverse cellular Activities (AAA^+^) family of mitochondrial resident proteases, suggests that mitochondrial translational inhibition may also be a feature of the UPR^mt^ pathway.

Mitochondria are the primary source of reactive oxygen species, and oxidatively damaged mitochondrial proteins are prone to misfolding and aggregation^1^. Moreover, four of the five mitochondrial respiratory chain complexes are composed of subunits encoded in both the nuclear and mitochondrial genomes, and an imbalance in the stoichiometry of these subunits can result in the accumulation of unfolded mitochondrial proteins^2^. Fortunately, mitochondria possess a pathway known as the mitochondrial Unfolded Protein Response (UPR^mt^) that confers protection from misfolded and aggregated proteins^2,3^. Similar to the cytosolic and endoplasmic reticulum (ER) UPR pathways, the UPR^mt^ results in the transcriptional activation of genes that promote the refolding or degradation of misfolded mitochondrial proteins^3^. Additionally, the accumulation of unfolded proteins in the ER and cytoplasm activates a kinase signaling cascade that triggers the inhibition of cytosolic translation in an apparent effort to limit the production of additional misfolded proteins during times of stress^4^. Under such conditions mRNAs become sequestered into large translationally inactive messenger ribonucleoprotein particles (mRNPs) known as stress granules^5^. However, previous work has not clearly established whether the accumulation of unfolded mitochondrial proteins triggers the activation of a pathway that leads to mitochondrial translational inhibition. In a recent article published in *Cell Death Discovery*, we provide evidence for the existence of such a pathway^6^.

To better understand mitochondrial proteostasis, we are studying the ATPase Associated with diverse cellular Activities (AAA^+^) family of mitochondrial resident proteases. We and others have used classical genetics, CRISPR/Cas9-mediated gene targeting, and RNAi to study the consequences of inactivating AAA^+^ proteases in the fruit fly *Drosophila melanogaster*^6–9^. In our most recent work, partial inactivation of the matrix-localized protease Lon caused decreased lifespan, locomotion defects, accumulation of unfolded mitochondrial proteins, and activation of the UPR^mt^ pathway^6^. Lon knockdown also reduced the abundance of all respiratory chain complexes that contain mitochondrial DNA encoded subunits. Further experiments indicated that this reduction was caused by an impairment of mitochondrial translation. The mitochondrial translation defect in Lon-deficient flies appeared to be caused largely by sequestration of mitochondrially encoded transcripts into dense ribonucleoparticles, which crudely resemble the stress granules that accumulate in the cytoplasm upon unfolded protein stress in the cytosol and ER^5^. Overexpression of another matrix-localized AAA^+^ protease, ClpP, partially rescued a behavioral deficit of Lon knockdown flies, suggesting that ameliorating the accumulation of unfolded proteins may rescue the translational defect. Together, these findings led us to conclude that UPR^mt^ activation reduces mitochondrial translation through a mechanism analogous to that of the cytosolic translation inhibition seen in other UPR pathways (Fig. 1).

**Fig. 1:**
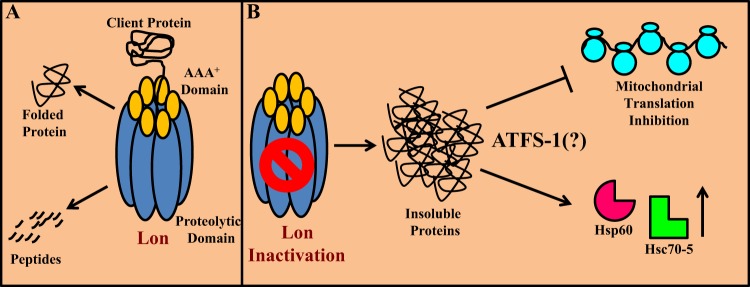
**a** Lon protease performs dual activities including functioning as a chaperone to refold misfolded proteins and as a protease to degrade misfolded proteins. **b** Inactivation of Lon protease results in the accumulation of misfolded proteins, thereby activating the unfolded protein stress response pathway and inhibiting mitochondrial translation. The mechanism of translation inhibition is unknown

While our work was under review, Zurita Rendón et al. reported the consequences of inactivating Lon protease in immortalized human skin fibroblasts^10^. Consistent with our findings, they found that Lon inactivation resulted in the accumulation of unfolded mitochondrial proteins, mildly increased expression of several UPR^mt^ components, and dramatically attenuated mitochondrial translation. In addition, they found reduced abundance of several mitochondrial ribosomal subunits upon Lon inactivation, leading the authors to attribute the translational defect to reduced ribosome biogenesis. However, the sedimentation properties of mitochondrially encoded transcripts were not explored in this study, so it remains possible that sequestration of these mitochondrial transcripts into large ribonucleoprotein complexes also contributes to the translational defect caused by Lon inactivation in human cell culture.

Previous work has shown that inactivation of other AAA^+^ family members can also result in mitochondrial translation inhibition through mechanisms that appear distinct from the mechanism we propose for Lon^11,12^. For example, inactivation of ClpP results in the accumulation of the ClpP substrate Eral1, a putative 12 S rRNA chaperone, which is believed to impair mitochondrial ribosome assembly^12^. Afg3L2 has also been shown to promote maturation of the mitochondrial ribosomal protein MrpL32, and inactivation of Afg3L2 impairs mitochondrial ribosome assembly and mitochondrial translation^11^. However, these studies have not ruled out the possibility that a novel arm of the UPR^mt^ pathway also contributes to the attenuation of mitochondrial translation upon Afg3L2 and ClpP inactivation. Moreover, our proposal that unfolded mitochondrial proteins trigger translational inhibition as part of the UPR^mt^ is strengthened by a recent report demonstrating decreased mitochondrial translation upon chemical inhibition of the mitochondrial chaperone Trap1 in vertebrate cell culture^13^.

The precise mechanism by which the UPR^mt^ may act to inhibit mitochondrial translation will require further investigation. In the nematode *Caenorhabditis elegans*, UPR^mt^ activation requires the transcription factor ATFS-1, but the mammalian ATFS-1 equivalent has only recently been identified^2,3,14^. Important goals of future research will be to identify targets of ATFS-1 that may be responsible for translational attenuation, and to test whether other perturbations that result in the accumulation of unfolded proteins and UPR^mt^ activation, such as mitochondrial chaperone inhibition, also cause attenuation of mitochondrial translation and accumulation of mitochondrial transcripts in dense ribonucleoprotein complexes. Our previous and ongoing studies of the *Drosophila* AAA^+^ mitochondrial proteases provide a foundation to further study the connections between unfolded mitochondrial protein accumulation, UPR^mt^ activation, and mitochondrial translational inhibition.
